# DE-CNN: An Improved Identity Recognition Algorithm Based on the Emotional Electroencephalography

**DOI:** 10.1155/2020/7574531

**Published:** 2020-08-08

**Authors:** Yingdong Wang, Qingfeng Wu, Chen Wang, Qunsheng Ruan

**Affiliations:** Informatics School of Xiamen University, Xiamen, Fujian, China

## Abstract

In the past few decades, identification recognition based on electroencephalography (EEG) has received extensive attention to resolve the security problems of conventional biometric systems. In the present study, a novel EEG-based identification system with different entropy and a continuous convolution neural network (CNN) classifier is proposed. The performance of the proposed method is experimentally evaluated through the emotional EEG data. The conducted experiment shows that the proposed method approaches the stunning accuracy (ACC) of 99.7% on average and can rapidly train and update the DE-CNN model. Then, the effects of different emotions and the impact of different time intervals on the identification performance are investigated. Obtained results show that different emotions affect the identification accuracy, where the negative and neutral mood EEG has a better robustness than positive emotions. For a video signal as the EEG stimulant, it is found that the proposed method with 0–75 Hz is more robust than a single band, while the 15–32 Hz band presents overfitting and reduces the accuracy of the cross-emotion test. It is concluded that time interval reduces the accuracy and the 15–32 Hz band has the best compatibility in terms of the attenuation.

## 1. Introduction

Identity systems are essential for security systems in many applications including payment systems, the Internet of Things (IoT), and health devices to protect personal data by verifying the identity of people. Moreover, these systems are often used in the process of human and machine interfaces. Conventional verification methods include setting a password or using a smart card for verification. However, these methods suffer from lots of problems, including forgetting or even stealing the password. Subsequently, the verification method through the password has been gradually replaced by the biometric verification system in the last few years. The biometric verification system authenticates through biometric information. A biological information system includes preprocessing of physiological signals, machine learning, and pattern recognition. Then, the system compares these features with the database. Physiological and behavioural biometrics include the fingerprint, face pattern, gait model, and electrocardiography (ECG).

Although these biological systems are popular now, there are still many unresolved problems. More specifically, identification systems based on the face [1], iris [2], sound [3], and the fingerprint [4] recognition can be deceived by high-quality images, sound, and feature extractions, respectively. Moreover, since fingerprints abundantly remain on ordinary surfaces, they can be easily abused by malefactors.

ID recognition method based on biometric, electroencephalography (EEG) provides more choices for identification [5]. Many scholars have focused on the EEG wave because it consists of invisible and untouchable electrical neural oscillations. Therefore, the EEG wave is highly attack-resilient and cannot easily be deceived. Moreover, the EEG is affected by the style of thinking, mood, and even the atmosphere so that it is a unique wave [6]. Benefiting from the deep learning (DL) techniques in various applications such as insomnia diagnosis, seizure detection, sleep studies, emotional recognition, and brain-computer interface (BCI) [7–10], the accuracy of the EEG-based ID recognition has been remarkably improved. In order to ensure that EEG can be used for the identification, most studies focus on the design of experimental paradigms. For example, eye closing [11], visual stimulation [12], and multiple mental tasks [13] have been investigated and mentioned in this regard. Wu et al. [14] proposed the eye blinking and self- or non-self-rapid serial visual presentation, and then, they extracted features from the EEG and the eye blink. Finally, they applied a fusion technology with two features to obtain the final high estimation score. The average accuracy rate of the study cases reached 97.60%, the false acceptance rate (FAR) was 2.71%, and the false rejection rate (FRR) was 2.09%. Kang et al. [15] used an open-access motion-image EEG database for identification. They conducted the network analysis based on the phase synchronization and extracted 10 single-channel features and 10 multichannel features, and then, they calculated the Euclidean distance between each possible pair of row vectors in the training and validation data matrices. Finally, they found thresholds of different features and gained equal error rate (EER) and FRR when the FAR is set to 1%. They found that the EER for the Romberg test, eyes open (REO), and the Romberg test, eyes closed (REC), are 0.73% and 1.80%, respectively. Moreover, they showed that the FRR with 1% FAR for REO and REC is 1.10% and 2.20%, respectively. Sun et al. [16] used the biggest motion imagination EEG dataset. They applied the1D-convolutional long short-term memory neural networks to identify 109 subjects, where the best result was about 0.0041in terms of EER. Furthermore, Moctezuma et al. [17] utilized the imaginary speech EEG for 27 subjects and performed 33 repetitions of five imaginary words in Spanish and gained an accuracy of 97%.

Although reviewed models in Table 1 yield high precision results, it is a challenge for the brain to reproduce the same EEG. For example, Wu et al. [14] showed that as time passes by, people gradually became accustomed to the faces of strangers so that reproducing the original visual impact from the recorded data becomes difficult [19].

So, the question is how the specified content impact identity? Reviewing the literature indicates that few studies have been performed on the identity authentication of the EEG based on different stimuli. Zhang et al. [9] used the emotional EEG for the identification and found that the emotion has no impact on the identification of 12 s EEG. However, the method robustness for different emotions was not proved. The present study intended to select the SEED dataset, a public emotional EEG dataset, to eliminate the impact of different contents on the brain by watching long videos. It is believed that only during watching the video, underlying characteristics and rhythms of the individual can be discovered.

Many studies have investigated the acquisition of rhythm features from the EEG. Kang et al. [15] extracted 10 multichannel features and 10 single-channel features, including seven spectra, and three nonlinearities, based on the phase synchronization network analysis for the subject identification. The performed analysis showed that Maxlyp has outstanding results compared with other features. Shi et al. [20] proposed the differential entropy (DE) of the EEG-based alert estimation and applied the proposed method to measure the complexity of EEG signals. Further studies [21, 22] showed that DE is a suitable scheme for emotional decoding. Moreover, Moctezuma et al. [17] applied the power spectral density (PSD) and autoregressive (AR) model coefficients for classification and obtained 99.76% accuracy in the studied cases. For the deep learning method, [18] applied the emotional EEG independent of any conventional features to predict the ID and achieved an accuracy of 94%. Therefore, the remaining question is “how to select the most suitable features and classification method for the model.”

In order to solve the problem, the computational expenses of features are initially compared. In this regard, two features are selected for the classification. Then, a novel preprocesses algorithm is proposed and the corresponding data is cut into small clips so that four-band data is obtained and each band's features are calculated. Finally, the model is designed with all features and the identification starts. In summary, the SEED dataset is utilized in the present study, which shields the correlation between the EEG and the specific content to find stable rhythm and characteristics in the EEG. Moreover, a novel method is proposed, which gains the algorithm accuracy higher than that of the algorithms. The present study contains three main highlights as the following:

- Compared to other algorithms, our algorithm takes less computational expense, while it has higher accuracy compared with conventional algorithms
- The proposed method is better compatible with different emotions than other methods
- For the first time, the factor of the EEG authentication interval was considered, which proved to be a strong attenuation with our algorithm

The rest of the article is organized as follows. The literature review for designing extraction features and deep learning-based EEG biometrics are presented in Section 2. Moreover, the detailed information and methodology of the proposed EEG biometric identification system are discussed in Section 3. Then experimental results and evaluations are presented in Section 4. Discussions on the performance and the corresponding potentials are provided in Section 5, which is followed by conclusions and a brief description of future works in the final section.

## 2. Related Work

### 2.1. Emotion and EEG

Various psychophysiology studies have demonstrated the correlations between human emotion and EEG signals [23, 24]. Martini et al. [25] noticed an increase in P300 in the late positive potential and an increase in gamma activity during viewing unpleasant pictures when the comparison is made with neutral pictures. Zhang and Lu [26] showed that energy from the beta and gamma bands increases in positive emotions and decreases in neutral and negative emotions. Moreover, neuroscience studies [27, 28] showed that EEG alpha bands reflect attention processing, while beta bands reflect emotional and cognitive processing in the brain. Considering the latest improvements in the emotional classification, the ACC has reached about 91% for recognizing a single person and 86% for recognizing different persons [29, 30]. Therefore, different emotions affect the EEG so that the issue of emotional robustness should be addressed prior to applying the EEG as an ineffective and reliable authentication method. According to [31], the recorded EEG rhythms can be categorized into five different rhythms in accordance with their frequency ranges. These rhythms, which are presented in Table 2, are as follows: delta (0.5–4 Hz), theta (4–8 Hz), alpha (8–15 Hz), beta (15–32 Hz), gamma (32–40 Hz), and the other bands (40–75 Hz). Delta wave always appears when people are in deep sleep. Moreover, the theta wave is encountered in early sleep stages and drowsiness. Alpha and beta rhythm are the typical rhythms during the relaxed state with closed eyes and the prominent rhythm during stressful situations, respectively. Finally, gamma rhythm is always involved in higher-order functions of the brain such as the feature binding of a perceived image. Therefore, the most suitable band can be explored for the identification.

### 2.2. Comparing Different Features

Kang et al. [15] demonstrated that three types of nonlinear EEG features, including the maximum Lyapunov exponent (Maxlyp), sample entropy, and the permutation entropy, have a higher impact on EEG-based biometrics than conventional spectral features. Application of the Maxlyp scheme can reach the best result at EER of 0.043 so that many researchers applied the different entropy methods on the EEG classification [32, 33]. Moreover, PSD is the most common feature in the EEG data. In the next section, it is intended to compare these three features from the time-consuming point of view.

#### 2.2.1. Maximum Lyapunov Exponent

Studies show that nonlinear methods, which mainly focus on the detection of characteristics of dynamic changes in a time series, are useful for clinical and scientific EEG applications [34, 35]. In the Maxlyp, the single-channel time series data *A*{*x*_1_,  *x*_2_,  *x*_3_,…*x*_*N*_} are considered, where *N* denotes the data length. In order to calculate the maximum Lyapunov exponent, the time series must be embedded into a D-dimensional space *x*_*j*_=[*x*_*j*_,  *x*_*j*−*t*_,   …,  *x*_*j*+(*D* − 1)*τ*_].

The Lyapunov exponent characterizes the inherent instability of a time series by quantifying the average rate at which nearby trajectories in the phase space diverge or converge [22, 36]. This instability is based on the sensitive dependence on the initial conditions. For two initial points in the phase close to each other space *X*_*j*1_ and *X*_*j*2_, *δ*_0_ is defined as the distance between points in the phase space ‖*X*_*j*1_ − *X*_*j*2_‖*σ*_0_ ≪ 1, where the distance varies to *σ*_Δ*n*_ after a certain time Δ*n*. ‖*X*_*j*1+Δ*n*_ − *X*_*j*2+Δ*n*_‖=*σ*_Δ*n*_. The correlation between *σ*_0_ and  *σ*_Δ*n*_  can be expressed in terms of an exponential function as follows:

(1) $$\begin{array}{c} \delta_{\Delta n\;}≅\delta_{0\;}\cdot e^{\lambda \Delta n}\left(\Delta n\gg 1,\;\delta_{\Delta n}\ll 1\right), \end{array}$$

(2) $$\begin{array}{c} \delta_{\Delta n}=\underset{\Delta n⟶\infty }{\lim}\delta_{0}\cdot e^{\lambda \Delta n}. \end{array}$$

The constant term in the exponent *λ* describes the rate of change and can be expressed in the following form:

(3) $$\begin{array}{c} \lambda =\;\underset{\Delta n⟶\infty }{\lim}\frac{1}{\Delta n}\ln\frac{\delta_{\Delta n\;}}{\delta_{0\;}}. \end{array}$$

Every *X*_*j*_ has one *λ*, where the maximum *λ* is the maximum Lyapunov exponent.

#### 2.2.2. Differential Entropy (DE)

Differential entropy scheme is applied for EEG-based vigilance estimation to measure the complexity of EEG signals [20, 37]. The DE scheme is mathematically expressed in the following form:

(4) $$\begin{array}{c} h\left(x\right)\;=\int_{x}f\left(x\right)\log\left(f\left(x\right)\right)dx, \end{array}$$

where *X* is a random variable and *f*(*x*) denotes the probability density function of  *X*. For series with the Gauss distribution (*N*(*μ*, *σ*^2^), the corresponding differential entropy can be expressed as

(5) $$\begin{array}{c} h\left(x\right)=\int_{-\infty }^{+\infty }\frac{1}{\sqrt[{2}]{2\pi \sigma^{2}}}\;e^{\left({\left(x-\mu \right)}^{2}/2\sigma^{2}\right)}\log\frac{1}{\sqrt[{2}]{2\pi \sigma^{2}}}e^{\left({\left(x-\mu \right)}^{2}/2\sigma^{2}\right)}dx=\;\frac{1}{2}\log\left(2\pi e\sigma^{2}\right). \end{array}$$

#### 2.2.3. PSD Subheadings

The periodic method PSD estimation is employed to simply find the discrete-time Fourier transform and scale the amplitude value of the result. In this scheme, *L* is defined as the signal *x* (*n*) length and *F* is the sampling frequency, respectively. In fact, the PSD value should be calculated at point *N*. The periodic estimation of the PSD method is expressed as follows:

(6) $$\begin{array}{c} \text{psd}=\frac{1}{\text{LF}}{\left(\sum_{n=0}^{L-1}\frac{x\left(n\right)e^{-2\pi jf\left(n\right)}}{\text{F}}\right)}^{2}. \end{array}$$

Table 3 shows a comparison of the three features and indicates that although the Maxlyp is the best feature, it consumes 12.591 s for calculation, where such high time consumption cannot be justified. Meanwhile, the complexity of the PSD and ED schemes is lower than that of the Maxlyp scheme.

### 2.3. Normalization

The EEG signal with *N* Channels is applied as the input for training the proposal neural network. When all the clips of DE values are obtained, the normalization overtime is required for each channel. The normalization is conducted as follows:

(7) $$\begin{array}{c} I_{i,j}=\left(\frac{{\text{DE}}_{i,j}-\left(\sum_{j=1}^{N}{\text{DE}}_{i,j}/N\right)}{\sigma_{i}}\right), \end{array}$$

where *i*, *j*, and *σ* refer to the signal position, channel position, and the standard deviation of the DE at one position of the DE sequence, respectively.

### 2.4. Changing Data from 1D to 2D

**Figure 1:**
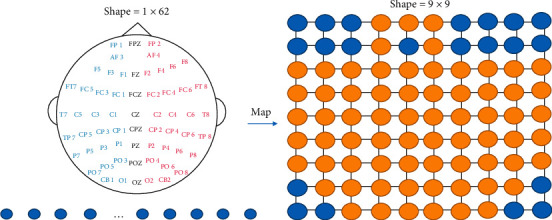
Schematic diagram of converting one-dimensional data into two-dimensional data.

The EEG-based BCI system uses a wearable headset with multiple electrodes to capture EEG signals. The International 10–20 System is an internationally recognized method of describing and applying the location of the scalp electrode and the underlying area of the cerebral cortex. It should be indicated that “10” and “20” numbers indicate that the actual distance between the adjacent electrodes is either 10% or 20% of the total front-back or right-left distance of the skull [38]. Although all positions in the data are meaningful, the sample EEG data is still a sequence after DE features are computed so that they are organized from the left to the right. In order to obtain the spatial features, data is converted into two-dimensional data in the manner of Figure 1. When there is no signal in the matrix, it will be replaced with zero.

## 3. Materials and Methods

### 3.1. Data

To develop the algorithm for EEG-based biometrics, the SEED^1^ database [26] is utilized in this section, which is the largest publicly available database for emotional EEG. In this datasheet, 62-channel EEG signals are recorded from fifteen persons when they are watching fifteen 4-minute emotional video clips. EEG data of each person is recorded three times in different weeks, where each time contains fifteen sessions.  Table 4 presents the distribution of labels per person for three kinds of emotions, including the neutral, positive, and negative emotions. The downsampling on data is performed with the frequency of 200 Hz, where the 0–75 Hz filter is applied.

#### 3.1.1. System Overview

**Figure 2:**
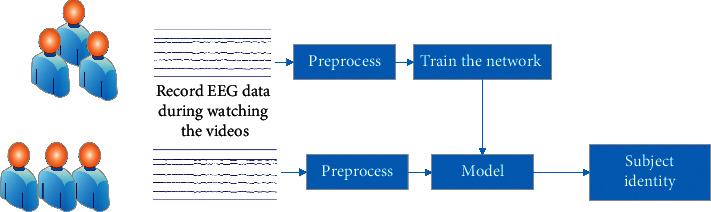
Overview of EEG-based identification.

Figure 2 shows an overview of the proposed EEG-based identification system consisting of the training period and the identification phase. In this system, the EEG features of all users are learned and stored in the DE-CNN model in the training period. It should be indicated that the EEG data, either in the training phase or in the identification phase, are preprocessed. The conducted preprocessing consists of segmenting data into 1000-sample length, computing the DE feature, and normalizing prior to feeding into the CNN model. The identification phase is the result of three CNN layers and a fully connected (FC) network with a Softmax activation function. In the rest of the methodology section, data segmentation, computing DE features, normalization, and multichannel CNN model will be described in detail.

#### 3.1.2. Preparing the Dataset

In the SEED dataset, the emotional EEG of each person is recorded three times. In the present study, two of three records are considered as the research data, while the last record will be tested for other purposes.  Table 4 indicates that there are three kinds of emotional states in the data, and each affection has the same number five of EEG trails. Wilaiprasitporn [39] used 12 s long emotional EEG as the test data and proved that the emotion does not affect the result so that the EEG data can be used for the identification. It is intended to explore a method to achieve less identification time. In this regard, the identification time is set to1000 samples (five-second × 200) to reflect the thinking rhythm. Thus, each EEG trial is simply segmented into 48 subsamples so that 720 subsamples (48 subsamples × 15 trials or clips) are obtained for each participant for one record. In summary, experiment labels are participant IDs. The data and labels of the present study can be described as follows:

1. Data: 2 × 15 × 720 × (1000 × 62)
2. Label: 2 × 15 × 720 × 1

#### 3.1.3. Preprocessing

**Figure 3:**
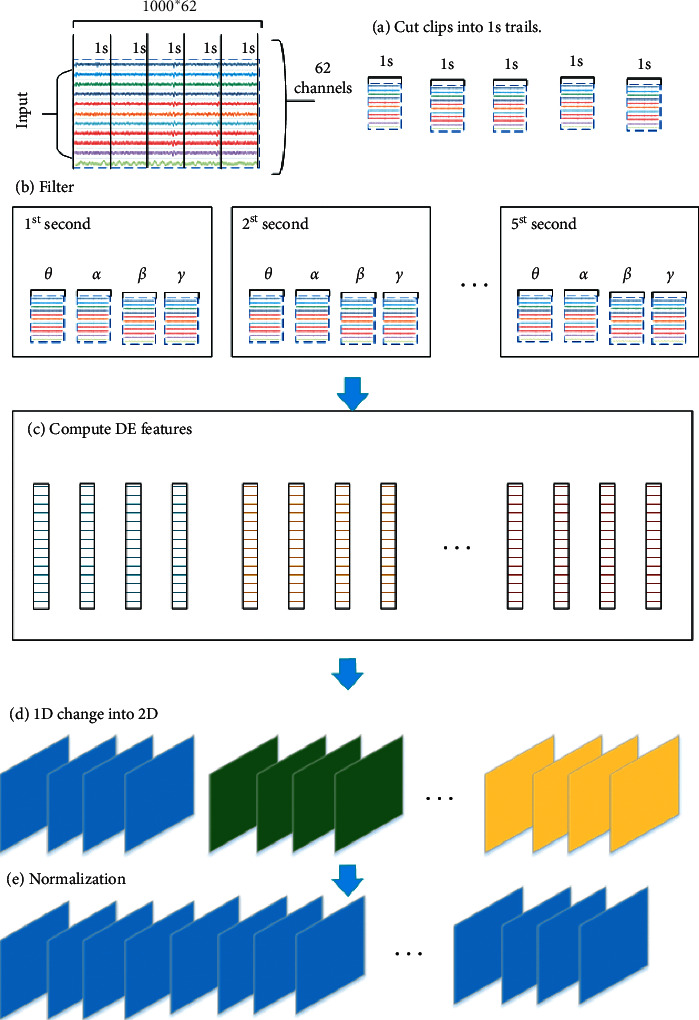
Main steps of the flow preprocessing: cutting, decomposing, computing, mapping, and normalization.

Figure 3 shows that the main flow of the preprocessing has five steps. In Step 1, in order to obtain the fine particle characteristics, the data is divided into five one-second sequences. Moreover, the features are extracted separately from sequences for finally being merged. In Step 2, decompose the EEG signal into four frequency bands (*θ*, *α*, *β*, *γ*) by the Butterworth filter, which has been proved to be more useful than the whole data in many kinds of research [18]. After decomposition, one clip EEG data is converted from 5 × 200 × 62 to 5 × 200 × 4 × 62. In Step 3, DE is an excellent feature and it is the output of a chaotic degree of sequence. Therefore, after computing every bands' channels DE features, the data is converted to 5 × 1 × 4 × 62. In Step 4, for the purpose of preserving spatial information among multiple adjacent channels, the one-dimension DE feature vector of 62 lengths to the 2D plane (9 × 9) is transformed according to the electrode distribution map. So, the data size is 5 × 4 × 9 × 9. In Step 5, in order to speed up the convergence of the model, normalization is performed at the end. This preserves all the information to the utmost.

#### 3.1.4. Convolution Neural Network

**Figure 4:**
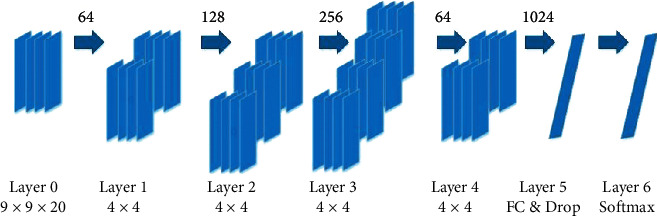
The classifier model for four layers. The number of 4 × 4 kernel for each layer is 64, 128, 256, and 64.

Figure 4 shows that a continuous convolution neural network with four convolution layers is used to extract features from the input cube. Moreover, a fully connected layer with dropout operation is added for the feature fusion and the Softmax layer is used for final classification. It should be indicated that there is no pooling layer between two adjacent convolution layers. In each convolution layer, zero-padding is applied to prevent information missing at the edge of the cube. More specifically, in the first three convolution layers, the kernel size is set to 4 × 4 and the stride is set to one. After the convolution operation, the RELU activation function is added to endow the model with nonlinear feature transformation capability. The first convolution layer with 64 feature maps is initiated and the feature maps in the following two convolution layers are doubled. Therefore, there are 128 and 256 feature maps in the second and third layers. In order to fuse different feature maps and reduce the computational cost, a one-by-one convolution layer with 64 feature maps is added. After these 4 continuous convolution layers, a fully connected layer is added to map the 649 × 9 feature maps into a final feature vector *f* ∈ 1024. Then, the following Softmax layer receives *f* to predict the human emotional state.

## 4. Results and Discussion

### 4.1. Comparison

Table 5 shows the comparison results of the proposed method and the state-of-the-art EEG-based identification methods. Among the mentioned methods, two deep learning methods exist. The first one is introduced in [9], which applies CNN + STML to classify five kinds of motor imagination and the accuracy of this method can reach 99%. Moreover, in [40], the authors applied the same method to explore the emotional effects on the identification with the same method in the DEAP dataset. The obtained result can reach an accuracy of 95% for 12 s. In order to compare this method with the proposed method of the present study, parameters are adjusted to suit the dataset of this study. In CNN + LSTM, three layers of 2D CNNs with 3 × 3 kernels are used. The number of filters starts with 128 in the first layer and continues with 64 and 32, respectively. It should be indicated that ReLu nonlinearity is used. Batch normalization and dropout are applied after every convolution layer. For the recurrent layers, 2 layers with 32 and 16 recurrent units are used, respectively. Moreover, recurrent dropout is applied. The dropout rates in each part of the model are fixed at 0.5. The RMSprop optimizer is used with a learning rate of 0.0005 and a batch size of 30.

Furthermore, 1D-convolution [16] applied the 1D-convolution long short-term memory neural network for the EEG-based user identification. The same parameters are applied in the present study for the proposed dataset. It is found that the model is different to converge. Therefore, three layers of 1D-convolution are used and the kernel sizes are 128, 256, and 512 with a dropout. Then, the results feed for the next two layers of LSTM with a kernel of 192. Finally, a dropout and a fully connected network with Softmax activation are applied to predict the probability of ID. The features of the last two methods are selected manually. Moreover, the same framework algorithm is compared based on the PSD. In the preprocessing, the average of the PSD is used in one second instead of DE. However, the other parts are the same as the DE-CNN.

In all experiments, the training, validation, and testing results are obtained by 10-fold cross-validation. It should be indicated that 90% of data are used as training and the left are used as the test dataset. The train time is the total time of computing one epoch at NVIDIA GEFDRCE RTX 2080ti. Table 5 shows that the 1D-LSTM cannot find enough information for the identification or overfit for the test dataset. Although CNN + LSTM has high accuracy, equal error rate (EER), it needs 278 seconds to train 32,361,999 parameters in one epoch. When the system adds a new user, it may take much time to update. The method proposed in the present study obtains the higher rank-1 of 0.997 and the EER of 0.00184 and it suits the high-demand security systems. Although the PSD is one of the most common used features, the result for PSD-CNN is lower 0.93 than that of the DE-CNN. The code for all comparison algorithms can be found in https://github.com/heibaipei/DE-CNN.

### 4.2. Comparison of Affective EEG-Based ID among Four Bands

**Figure 5:**
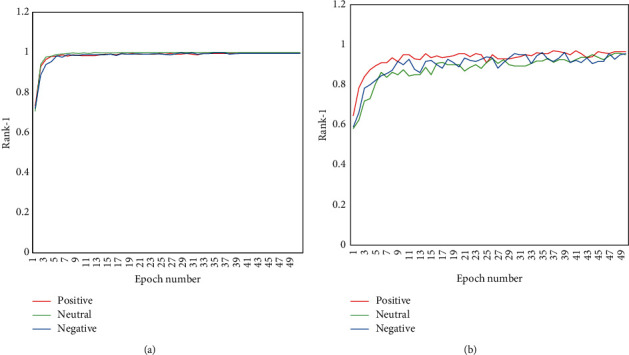
Training curve of the identity recognition based on three emotions for different algorithms: (a) DE-CNN algorithm and (b) CNN-LSTM algorithm.

In order to explore the best band to reduce noise, only two methods within four bands are compared in a positive mood.  Figure 5 shows the obtained results. All parameters are the same as the above-mentioned parameters. The number of training epoch is 50. Rank-1 with the *β* band and the 4–40 Hz band are a little higher than other bands, while the 4–40 Hz band is lower than the 14–31 Hz band. It is explicable that the beta band is highly correlated with attention and alertness. Moreover, it should be indicated that the wider band may have more noise.

**Figure 6:**
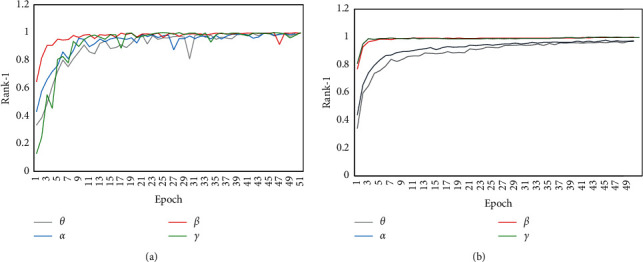
Testing curve of the identity recognition based on different bands: (a) results of the CNN-LSTM algorithm and (b) results of DE-CNN algorithm.

It is found that the obtained results of the two methods in 14–31 Hz and 4–40 Hz have little difference in the final accuracy, while the processes of the training have a significant difference. The results of the DE-CNN in four bands are smoother and faster than the CNN-LSTM.  Figure 6 shows the process of training. It should be indicated that beta waves work best in identity authentication consistent with the content of Table 6.

### 4.3. Comparison of Affective EEG-Based ID among Three Affective States

Many people have questioned the performance of the EEG for identity authentication. Moreover, the stability of the EEG-based method has attracted many scholars. Since different moods make different EEGs, it is of significant importance to train a robust model.  Table 7 shows that three emotions have little impact on the identification with DE-CNN, where the currency and EER approach 0.99 and 0.001, respectively, while applying the CNN-LSTM to the neutral emotion yields the worst result, where the corresponding EER is only 0.06.  Table 7 shows that two methods with different emotional EEG datasets almost have the same results at the end of the train. In fact, there is a little effect on rank-1 between three emotions. The left side of Figure 5 shows the test result of DE-CNN, while the right side shows the results of the CNN-LSTM. It is observed that the training process is more stable than the CNN-LSTM, while the DE-CNN convergences more quickly. In order to test the stability effect on the identification based on different moods, one of the affective models is utilized to predict the identity of the other two emotional states. It should be indicated that all methods are tested using default settings. Since performing all tests is not enough to find the most robust emotion, the one emotional model is also applied to the other two emotions.  Table 8 shows the results obtained from the sympathetic EEG-based ID detection. Neu-pos and Neg-pos represent neutral and negative emotional data testing based on the positive model, respectively. Moreover, Pos-neu and Neg-neu represent positive and negative emotional data testing based on the neutral EEG dataset, respectively. Furthermore, Pos-neg and Neu-neg denote positive and neutral emotional data testing based on the negative EEG dataset, respectively. Comparing the results of identity authentication for two different bands, DE-RNN is better than CNN + LSTM. It is also observed that different emotions affect the identification and reduce rank-1 of the identification. Comparing the results of two methods indicates that positive emotions have a larger impact on identity authentication than other emotions. In other words, the positive model is easier to overfit. It is found that neutral emotions are more robust to each other. Therefore, when the identification system based on the EEG is set up, the neutral mood is proposed. Considering both the CNN-LSTM and the DE-CNN methods, beta waves (15–32 Hz) have worse results compared with 0–75 Hz. When robustness is required, the model needs wider EEG frequency band.

### 4.4. Discussion

#### 4.4.1. Results of the Separated Dataset for the Identification

**Figure 7:**
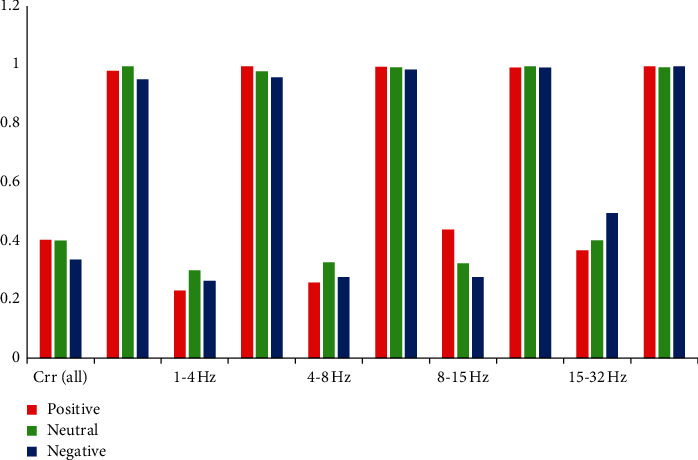
Rank-1 of the interval and noninterval verification. The graph in front of each band represents the interval result, while the latter represents the noninterval result. Red colour represents positive emotions, while green and blue colours represent neutral and negative emotion.

In the SEED dataset, each subject performs three times of EEG signal collections with different time intervals. The longest time interval is four months, while the shortest time interval is three days. Each of the first two experiment EEG signals is used as the training dataset, while the last experimental EEG signal is used as the test dataset. Then the length of the longest interval recommended for the identification is recorded. Moreover, the best compatible classification bands will be explored. It is found that EEG from different periods has different rank-1, where the details are presented in Table 9 and Figure 7. It indicates that the time interval rank-1 is lower than the noninterval. It should be indicated that NT represents the first two datasets of test rank-1, while the interval represents the last dataset accuracy based on the former train model. Moreover, different bands affect accuracy, where the 15–32 Hz band gives the best results. When time intervals of accuracy are obtained from the table, it is found that as the frequency increases, the results deteriorate. In this section, results are not compared with 32–75 Hz bands because the high frequency rarely appears in healthy people.

## 5. Conclusions

In order to evaluate the identity authentication based on the EEG, it is necessary to confirm that each person has a unique brain rhythm. In the present study, human EEG based on different contents is employed to recognize the identity. It is found that the proposed method can recognize the identity accurately.

Currently, there are two paradigms in processing the identification, where details are presented in Table 10. In the first paradigm, subjects see the same induced stimulus, where it reflects the different cognitive basis of a person. In the second paradigm, subjects see different contents induced stimuli, where longer EEG of each person should be divided into different parts to eliminate the variations caused by different contents. Personal characteristics are extracted during personal identification. There are many similarities between the brain wave and voice wave verification. Two main methods are proposed in this regard. In the first method, the extracted acoustic features are initially aligned with specific sounding units, features are projected into a lower linear space, and then speaker information is mined. Intuitively, “the difference between different people in the same tone [41–44]” can be understood as mining. The first method draws on some phonological knowledge that uses a vocal unit classification network for speech recognition. However, the second method “end-to-end deep learning-based authentication” is a purely data-driven approach. Through massive data samples and very deep convolution neural networks, the machine automatically explores speaker difference information in acoustic features to extract speaker information representations in acoustic features.

More specifically, the deep convolution neural network is trained by a large amount of voice wave data, and the output category is defined as the speaker ID. In actual training, tens of thousands of IDs are required for network training. Thus, a basic network capable of effectively characterizing the speaker is obtained. However, EEG is not easy to obtain a large number of samples due to equipment limitations. But EEG identification offers more choices for identity authentication.

The second most important feature in identity authentication is stability. In the present research, the second method is performed for identity authentication. In terms of stability, emotional factors and time factors are mainly considered. In the emotional factors, it is found that different emotions have little impact on the classification results with DE-CNN. While positive emotional robustness is the worst in terms of mutual migration, the neutral emotional robustness is best in three emotions. From the perspective of different frequency classification results, the frequency band of 15–32 Hz is superior to other frequency bands in both classification and migration. In terms of interval dimensions, the 15–32 Hz band is more compatible than the other bands of the EEG and performs well at different points during the time.

Based on our current research, we will explore cross identity identification methods next and design models to eliminate the impact of time on individual EEG, which is very important, and this is the key point for EEG to become a daily identity authentication.

## Figures and Tables

**Table 1:** Summary of the literature review.

Papers	EEG content	Method	Time (s)	EER	AAC
[14]	Eye blinking and self- or non-self-rapid serial visual presentation	Machine learning	3	—	0.9076
[15]	Relax and eye-closed	Machine learning	60	0.0073	0.9893
[16]	MI-EEG	1DCNN-LSTM	1	0.0041	0.995
[17]	Imaginary speech	Deep learning	Four words	—	0.97
[18]	Relax and eye-closed	Attention-RNN	1/128	—	0.998
[9]	Emotion video (different stimulant)	2DCNN + LSTM	12	—	1

**Table 2:** The range of applications for different waves.

Rhythm	Frequency domain	Brain states	Awareness degree
Delta	0.5–4 Hz	Deep dreamless sleep	Lower
Theta	4–8 Hz	Creative, intuitive, drowsy	Low
Alpha	8–15 Hz	Relax, not drowsy, tranquil, conscious, focus	Medium
Beta	15–32 Hz	Thinking, aware of self-surroundings	High
Gamma	32–40 Hz	Thinking, integrated thought	Very high

**Table 3:** The time of computing three features.

Feature name	Maxlyp	ED	PSD
Time (s)	12.591	0.00064	0.0019

**Table 4:** Number of emotions per person.

Label	Positive	Neutral	Negative	Totally
Number	5	5	5	15

**Table 5:** Comparison of the proposed method with some state-of-the-art EEG-based identification methods.

Name	Train time (s)	Rank-1	Parameters	EER
CNN + LSTM [9]	278	0.98 ± 0.002	32361999	0.0121
D-LSTM [16]	60	0.76 ± 0.04	1060943	0.011
DE-CNN	2	0.997 ± 0.0028	7918095	0.002
PSD-CNN	2	0.934 ± 0.0034	7918095	0.021

**Table 6:** Results of identification based on the four EEG bands.

Rank-1	4-7 Hz	8–14 Hz	14–31 Hz	32–40 Hz	4–40 Hz
CNN + LSTM	0.986	0.959	0.997	0.978	0.993
DE-CNN	0.864	0.972	0.998	0.994	0.996

**Table 7:** Rank-1 and EER of identification in three emotions.

	CNN + LSTM			DE + CNN		
Label	Positive	Neutral	Negative	Positive	Neutral	Negative
Rank-1	0.97	0.91	0.969	0.996	0.996	0.995
EER	0.012	0.06	0.01	0.001	0.001	0.002

**Table 8:** Rank-1 of cross-emotion verification on identity recognition.

	CNN + LSTM		DE + CNN	
Rank	0–75hz	15–32hz	0–75hz	15–32hz
Neu-pos	083	0.95	0.915	0.83
Neg-pos	0.83	0.91	0.883	0.81
Pos-neu	0.89	0.84	0.91	0.914
Neg-neu	0.92	0.95	0.952	0.948
Pos-neg	0.86	0.78	0.897	0.845
Neu-neg	0.906	0.93	0.969	0.973

**Table 9:** Rank-1 of time interval emotion classification results.

DE-CNN	0.5–75 Hz		1–4 Hz		5-8 Hz		9–15 Hz		15–32 Hz	
Rank-1	Interval	NT	Interval	NT	Interval	NT	Interval	NT	Interval	NT
Positive	0.404	0.98	0.23	0.995	0.258	0.994	0.438	0.991	0.367	0.996
Neutral	0.4	0.996	0.3	0.978	0.326	0.992	0.324	0.995	0.402	0.993
Negative	0.336	0.952	0.264	0.957	0.276	0.984	0.276	0.991	0.495	0.995

**Table 10:** Two ID recognition paradigms.

Paradigms	Content	Interpretability	Time validity
1	Same evoked content.	Strong	Weak attenuation
2	Different evoked content or the same evoked content; the order is different.	Weak	Strong attenuation

## Data Availability

The SEED data used to support the findings of this study are included within the article.
